# Rupture of Hematoma of the Broad Ligament—Operated

**Published:** 1901-02

**Authors:** B. Dennis


### Clinical Report.

### RUPTURE OF HEMATOMA OF THE BROAD LIGAMENT—OPERATED.

By Dr. L. G. HANLEY,
Gynecologist, Buffalo Hospital of the Sisters of Charity.

Reported by B. DENNIS, M. D.

MRS. B., age 34, married; number of children, two. Admitted to hospital, October 8, 1900. Patient was pulseless at wrist, breathing hurriedly, restless and in extremis, presenting all evidences of severe internal hemorrhage. Patient had not menstruated for five months and thinking she was pregnant, sent for her family physician to exmine her. During the examination she was seized with a severe pain, became weak, till she was in the condition above described. This occurred fourteen hours before being admitted to hospital. Strychnia and brandy were given, pro re nata, by the family physician.

The ordinary preparation given, and abdomen opened, when about two quarts of clotted blood was removed from the pelvic cavity. The uterus was drawn up through the opening, which showed extensive bleeding from broad ligament of right side. Ligament clamped and all attention directed to stimulating patient by injections of normal salt solution under the breast—strychnia, brandy, etc. Abdominal cavity flushed with saline solution, when patient rallied sufficiently for completion of work. There was a tear one and one half inches long, extending down from right cornu of uterus, which was sutured in order to check hemorrhage from wound of serosa. The wound in ligament closed after sac was removed. Abdomen filled with normal salt solution. The ordinary surgical technique was observed. Patient left hospital in twenty-eight days.

One and one half years ago hysteropexy was performed by intra-abdominal shortening of round ligaments. Examination showed no evidences of fetus or chorionitis villi, though she had not menstruated for five months.

---

The constitutional treatment in chronic inflammation will depend upon the disease which is at the bottom of the process, rather than on the process itself.—*Cheyne and Burghard.—Railway Surgeon.*

---

Free incisions into an inflamed part will often prove of the very greatest value in acute inflammatory conditions.—*Cheyne and Burghard.—Railway Surgeon.*

